# Root mucilage enhances plant water use under combined soil and atmospheric drought

**DOI:** 10.1093/aob/mcaf182

**Published:** 2025-08-13

**Authors:** Asegidew Akale, Mohanned Abdalla, Tina Koehler, Anna M Sauer, Efstathios Diamantopoulos, Mutez A Ahmed

**Affiliations:** Root–Soil Interaction, School of Life Sciences, Technical University of Munich, Freising, 85354, Germany; Root–Soil Interaction, School of Life Sciences, Technical University of Munich, Freising, 85354, Germany; Department of Horticulture, Faculty of Agriculture, University of Khartoum, Khartoum North, 13314, Sudan; Root–Soil Interaction, School of Life Sciences, Technical University of Munich, Freising, 85354, Germany; Root–Soil Interaction, School of Life Sciences, Technical University of Munich, Freising, 85354, Germany; Soil Physics, University of Bayreuth, Bayreuth, 95447, Germany; Root–Soil Interaction, School of Life Sciences, Technical University of Munich, Freising, 85354, Germany

**Keywords:** Cowpea, vapour pressure deficit, soil drying, soil hydraulic conductivity

## Abstract

**Background and Aims:**

Plants have evolved various root adaptive traits to enhance their ability to access soil water in stressful conditions. Although root mucilage has been suggested to facilitate root water uptake in drying soils, its impact during combined edaphic and atmospheric stress remains unknown. We hypothesized that mucilage decreases the saturated soil hydraulic conductivity, and consequently, a genotype with high mucilage production will exhibit lower maximum soil–plant hydraulic conductance and restrict transpiration at relatively low vapour pressure deficit (VPD). On the contrary, in drying soil, mucilage attenuates the gradients in matric potential at the root–soil interface and thus facilitates root water uptake, especially at high VPD.

**Methods:**

We compared two cowpea genotypes with contrasting mucilage production rates and subjected them to three consecutively increasing levels of VPD (1.04, 1.8 and 2.8 kPa) while the soil was left to dry out. We measured the transpiration rate and soil and leaf water potentials and estimated canopy and plant hydraulic conductance during soil drying.

**Key Results:**

In wet soil conditions, the high-mucilage genotype restricted transpiration rate at lower VPD (1.46 kPa) compared with the low-mucilage genotype (1.58 kPa). Likewise, the initial slope of transpiration rate in response to VPD (the maximum conductance) was significantly lower in the high- compared with the low-mucilage genotype. During soil drying, the transpiration rate declined earlier in the low- compared with the high-mucilage genotype, supporting the hypothesis that mucilage helps to maintain the hydraulic continuity between roots and soil at lower water potentials in the high-mucilage genotype.

**Conclusions:**

Root mucilage is a promising trait that reduces water use in wet soil conditions, thereby conserving soil moisture for critical phases (e.g. flowering and grain filling), both on a daily basis (increasing VPD) and on a seasonal time scale (soil drying).

## INTRODUCTION

Drought stress in plants can occur when water availability is insufficient to meet the needs of the plants for water, resulting from a mismatch between water supply from the soil (soil drying) and atmospheric demand (atmospheric drying) (Humphrey *et al.*, 2021). Water flow across the soil–plant–atmosphere continuum is driven by a gradient in water potential. This negative tension enables plants to extract water from the soil, transports it through the stem and releases it into the atmosphere through transpiration. Although this long-distance water transport in plants is primarily passive, it can be influenced actively by modifying plant traits such as stomata function and plant hydraulic transport capacity (Taylor, 1964; Fricke, 2017; Ahmed *et al.*, 2018*a*; Torres-Ruiz *et al.*, 2024), which allows plants to adapt to changing environmental conditions (Tardieu *et al.*, 2018; Cai *et al.*, 2022).

Plant transpiration exhibits a diurnal pattern, being lowest at sunrise and increasing to a maximum around midday (Zhang *et al.*, 2017). Transpiration is predominantly governed by the evaporative demand [such as vapour pressure deficit (VPD)] and conductances along the soil–plant–atmospheric continuum, i.e. soil, rhizosphere, root, xylem and leaf hydraulic conductance (Carminati and Javaux, 2020; Abdalla *et al.*, 2021; Bourbia *et al.*, 2021). Stomatal conductance decreases with increasing VPD (Grossiord *et al.*, 2020) and the non-linear response of transpiration rate to increasing VPD is typically referred to as the VPD breakpoint (Devi and Reddy, 2018; Gholipoor *et al.*, 2013). It has been demonstrated that stomatal conductance decreases with increasing VPD, even in well-watered conditions (Bunce, 2006; Baca Cabrera *et al.*, 2020). Although the mechanisms governing stomatal aperture and closure have been studied extensively (Hetherington and Woodward, 2003), the processes driving the transpiration rate limitations to increased VPD remain contentious (Grossiord *et al.*, 2020). In angiosperms, this response is thought to involve diverse mechanisms ranging from passive change in guard cell turgor to hormonally controlled aperture linked to active sensing of water status within the leaf (Tombesi *et al.*, 2015; Grossiord *et al.*, 2020; Cai *et al.*, 2023; Scoffoni *et al.*, 2023; Binstock *et al.*, 2024). In the context of soil drying, the reduction in transpiration is believed to involve stomatal closure triggered by a decrease in below-ground hydraulic conductivity (Abdalla *et al.*, 2021, 2022; Koehler *et al.*, 2023; Manandhar *et al.*, 2024). Rodriguez-Dominguez and Brodribb (2020) and Abdalla *et al.* (2022) demonstrated experimentally that the drop in hydraulic conductance at the root–soil interface is the primary cause for the stomatal closure and thus transpiration reduction during soil drying. Higher VPD can exacerbate the speed of soil drying, thus modifying the plant drought stress dynamics. Likewise, soil drying will increase the stomatal sensitivity to increasing VPD (Cai *et al.*, 2024; Koehler *et al.*, 2024; Novick *et al.*, 2024). Hence, understanding the combined effect of atmospheric drought and soil drying is key to studying plant water use and its response to environmental stresses.

Plants have adapted various above- and below-ground traits to regulate water loss during periods of drought stress. Plants can limit the transpiration rate during high evaporative demand and early in the soil drying cycle through partial stomatal closure (Richards and Passioura, 1989; Sinclair *et al.*, 2005). Soil water conservation during the early growing season by limiting the transpiration rate during the periods of high VPD could potentially enhance both yield gain and stability, especially in water-limited environments (Richards and Passioura, 1989; Sinclair *et al.*, 2010; Choudhary *et al.*, 2014; Messina *et al.*, 2015). Restricting the transpiration rate under high VPD can be seen as an anticipatory process to deal with terminal drought conditions (Rodriguez-Gamir *et al.*, 2016; Moussa *et al.*, 2019). This strategy allows plants to conserve water even before the onset of water scarcity and maintain their vital functions longer during drought (Tardieu *et al.*, 2018). In contrast, below-ground traits that might influence plant water use include, for instance, the formation of root hairs (Cai and Ahmed, 2022) and the association with arbuscular mycorrhizal fungi (Abdalla *et al.*, 2023).

Another mechanism suggested to regulate water loss during periods of drought stress takes place at the root–soil boundary through mucilage exudation. Plants have a natural capacity to produce compounds that interact with the soil, supposedly enhancing water availability to the plants and retaining water in the soil (Bengough *et al.*, 2011; Naveed *et al.*, 2019; Berauer *et al.*, 2023). Mucilage is a polymeric gel secreted from the cap cells of the root tip in most plant species (Ahmed *et al.*, 2014; Jianga *et al.*, 2022). It comprises primarily polysaccharides, with minor components such as amino acids, organic acids, proteins, glycolipids and phospholipids (e.g. Nazari *et al.*, 2020; Werner *et al.*, 2022). Mucilage has been proposed as a means of facilitating root water uptake during soil drying (Ahmed *et al.*, 2014; Carminati *et al.*, 2016). Mucilage can absorb large volumes of water, altering the physical properties of the rhizosphere during soil drying (Ahmed *et al.*, 2016; Benard *et al.*, 2018; Zarebanadkouki *et al.*, 2019; Nazari, 2021). Furthermore, mucilage also alters the pore space and influences functional soil physical properties, such as water retention and hydraulic properties, thus helping to delay the onset of hydraulic discontinuity between root and soil (Ahmed *et al.*, 2014; Carminati *et al.*, 2017; Knott *et al.*, 2022) and enabling plants to maintain transpiration as the soil becomes drier (Abdalla *et al.*, 2024).

Although previous studies indicate that mucilage reduces the saturated hydraulic conductivity of the soil (Ahmed *et al.*, 2018*b*; Kroener *et al.*, 2018; Zarebanadkouki *et al.*, 2019) and facilitates root water uptake during soil drying (Ahmed *et al.*, 2014; Abdalla *et al.*, 2024), the effect of mucilage on combined soil and atmospheric drying remains unknown. We hypothesize that, in wet soil conditions, high mucilage production decreases the saturated soil hydraulic conductivity, leading to the adaptive development of a lower maximum soil–plant conductance and thus resulting in an earlier restriction of transpiration at a relatively low VPD. On the contrary, during soil drying, mucilage is expected to attenuate the drop in matric potential at the root–soil interface and extend the range of water potentials in which roots and soil remain hydraulically connected, hence maintaining transpiration in relatively drier soil conditions. Furthermore, we hypothesize that, during soil drying, the drop in matric potential around the root–soil interface could lead to a steeper decline in canopy conductance for a low-mucilage genotype compared with a high-mucilage genotype.

To test our hypotheses, we compared two cowpea (*Vigna unguiculata* L.) genotypes with contrasting mucilage production rates. Cowpea was chosen as a model plant because it is the only species in which we have genotypes with contrasting mucilage production, which helps us to examine the potential role of mucilage in water uptake in conditions of soil and atmospheric stress. We subjected the two genotypes to three consecutively increasing levels of VPD (1.04, 1.8 and 2.8 kPa) while the soil was left to dry out. During soil drying and increasing VPD, we monitored the transpiration rate (*E*), soil water content (SWC), leaf water potential (LWP) and soil water potential (SWP). We estimated the canopy conductance ($g_{c}$) and compared the two genotypes in wet soil conditions. We also estimated the plant hydraulic conductance (*K*_p_) from the relationship between transpiration rate and leaf water potential at a given soil water potential.

## MATERIALS AND METHODS

### Plant and soil preparation

We used two cowpea genotypes with contrasting mucilage production rates (Sun *et al.*, 2015). One cowpea genotype produces low amounts of mucilage and the other produces high amounts. According to Sun *et al.* (2015), the low-mucilage-producing genotype (Low mucilage) had 0.5 mg dry weight mucilage per gram of dry weight roots, whereas the high-mucilage-producing genotype (High mucilage) had a mucilage content of 3.4 mg dry weight mucilage per gram of dry weight roots. The seeds for those two genotypes were sterilized with 10 % H_2_O_2_ for 10 min and germinated on saturated filter papers in Petri dishes placed in darkness. Two days later, the germinated seeds (eight of each genotype) were transferred individually to PVC columns (9 cm in diameter, 30 cm in height) filled with sandy soil, a mixture of 16.7 % loamy soil by weight with quartz sand (83.3 %). The hydraulic properties of these soils were reported previously by Vetterlein *et al.* (2021) and are compiled in the Supplementary Data (Fig. S1). Before being placed into the columns, the soil was passed through a 1 mm sieve to create homogeneous soil layers among the replicates.

After germination, a layer of plastic beads (with a diameter of 3.5 mm) was applied to the top of the columns to minimize evaporation from the soil surface. To prevent soil warming, aluminium foil was used to wrap the sides of the transparent column, which had five holes (5 mm) on the side of the column walls to enable soil moisture measurements at different depths.

### Plant growth conditions

Eight plants of each genotype were grown in a controlled walk-in climate chamber (ThermoTEC Weilburg, Germany). During the daytime, throughout the growth period, the plants were exposed to three consecutive VPD levels. Low VPD (1.04 kPa) was set at 24.5 °C with a relative humidity (RH) of 68 %. The medium VPD (1.8 kPa) was produced with an RH of 50 % at 29.5 °C, and the high VPD (2.8 kPa) was set at 33.5 °C with an RH of 40 %. The duration of each VPD level was 3 h, with a 30 min transition period to simulate natural conditions (Supplementary Data Table S1). All VPD levels were set with an identical light intensity of 1100 µmol m^−2^ s^−1^. During the nighttime, plants were exposed to a temperature of 18.5 °C and RH of 78 % for 8 h. Plants were watered daily to ensure optimal water supply and to maintain growth until the start of measurements. The temperature and RH inside the chamber were monitored at canopy height every 10 min using a thermo-hygrometer (EL-USB-1, Lascar Electronics, UK), and VPD (in kilopascals) was calculated as follows.


(1) $$\mathrm{VPD}=\frac{1-\mathrm{RH}}{100}\times \frac{610.7\times 10^{\frac{\left(7.5T\right)}{\left(237.3+T\right)}\;}}{1000}$$

*T* is temperature (in degrees Celsius), and RH is relative humidity (as a percentage).

### Soil and plant hydraulic measurements

Data collection began 35 days after sowing, when most plants had reached the early flowering stage. At this point, irrigation was withheld, and pots were allowed to dry. Measurements took place during soil drying for 6 days after the last irrigation (see details below). Volumetric soil water content was monitored daily using a time domain reflectometer (TDR; E-Test, Lublin, Poland). The measurement was taken from five depths for all replicates, and the average value was considered as the soil water content. In addition, soil water potential was measured during soil drying using water potential sensors (TEROS 31; Meter Group, Munich, Germany).

After the last irrigation, the transpiration rate was obtained gravimetrically for all replicates after each VPD level. We weighed the columns manually at the beginning and end of each VPD level using a sensitive balance (Plattform Wägezelle H10A, Bosche, Germany) with a capacity of 8000 g and precision of 0.01 g. The transpiration rate was then calculated based on the changes in weight of the columns and the corresponding time duration for each VPD level. Before the dry-down experiment began, we measured the transpiration rate in well-watered conditions for two consecutive days using the same method. Plants received full irrigation each night, and transpiration rates were measured after each VPD level the following day to ensure that transpiration remained constant in well-watered conditions.

We measured the leaf water potential after each VPD level and at predawn every other day using a Scholander pressure bomb. Leaf water potential was measured for one leaf per plant, with four replicates per genotype.

Plant hydraulic conductance (*K*_p_; in grams per second per megapascal) was estimated from the measurement of *E*, SWP ($\psi_{\mathrm{soil}}$) and LWP ($\psi_{\mathrm{leaf}}$) in well-watered conditions. The value of *K*_p_ was calculated as follows:


(2) $$K_{p}=\frac{E}{\psi_{\mathrm{soil}}-\;\psi_{\mathrm{leaf}}}$$

Leaf area was determined at the end of the experiment. Following harvest, leaves of each individual replicate were imaged to determine the leaf area (in centimetres squared) using ImageJ software (Schneider *et al.*, 2012). The biomass, for all replicates, was reported as the dry weight after oven drying at 60 °C for 72 h (Poorter and Nagel, 2000). In addition, root biomass was measured in four replicates per genotype using the oven drying method. Root:shoot ratio was determined by pooling the shoot dry weight of four plants per genotype and matching it with the root dry weight of the same plants.

### Normalization of transpiration

To assess transpiration reduction after the last irrigation for each plant, transpiration rate values were normalized as follows: the transpiration rate of each day for each plant was divided by the average transpiration rate of the first 2 days when the plants were still at the well-watered stage. The daily normalized transpiration rate (NTR) for each sample was calculated as follows (as described by Devi and Reddy, 2020):


(3) $$\mathrm{NTR}=\frac{\;E\_day\_n}{\mathrm{mean}\;E\_days\_1-2}$$

Where *E_day_n* is the daily transpiration rate after last irrigation, and mean *E_days_1–2* is the average transpiration rate of the first 2 days when plants were at the well-watered stage.

### Assessment of fraction of transpirable soil water threshold in the drying conditions

The fraction of transpirable soil water (FTSW) is water that is available in the soil for plant transpiration. The FTSW was computed as the soil water content for that day (pot weight of the day minus pot weight at the end of the drying cycle) relative to the total transpirable soil water, which is the difference between the initial and final pot weights. Based on this calculation, the FTSW was estimated to be one when the pots were initially watered to pot water holding capacity (100 %) and zero when NTR falls below 0.1 (Sinclair and Ludlow, 1986):


(4) $$\mathrm{FTSW}=\frac{\mathrm{daily}\;\mathrm{pot}\;\mathrm{weight}-\mathrm{final}\;\mathrm{pot}\;\mathrm{weight}}{\left(\mathrm{initial}\;\mathrm{pot}\;\mathrm{weight}-\mathrm{final}\;\mathrm{pot}\;\mathrm{weight}\right)}$$

### Calculation of canopy conductance

Canopy conductance ($g_{c}$; in grams per second per centimetre squared), was determined according to Jarvis and McNaughton (1986) .


(5) $$g_{c}=\frac{E}{\mathrm{LA}}\times \frac{P_{\mathrm{atm}}}{\mathrm{VPD}}$$

Where $g_{c}$ is the canopy conductance, *E* is the transpiration, LA is the leaf area, *P*_atm_ is the atmospheric pressure (*P*_atm_ = 101.325 kPa), and VPD is the vapour pressure deficit.

### Statistical analysis

Segmental linear regression analyses were performed for each plant (i.e. eight replicates per genotype) to examine the transpiration rate in response to increasing VPD using the R segmented package v.4.3.2 (Muggeo, 2023). This analysis allowed us to identify the VPD breakpoints (VPD_BP_) and determine the initial slope (Slope1) and the slope after the breakpoints (Slope2) in the transpiration rate response to an increase in VPD. The same analysis was performed to calculate the fraction of transpirable soil water breakpoints (FTSW_BP_) threshold in NTR response to FTSW. Furthermore, the soil water content (in centimetres cubed per centimetre cubed), at which the normalized transpiration rate began to decline (the critical soil water content), was also determined using segmented linear regression analysis. Moreover, an independent *t*-test was performed to evaluate the significant difference between the mean of vapour pressure deficit breakpoint (VPD_BP_; the VPD at which the increase in *E* is restricted with rising VPD), Slope1, leaf area, dry shoot biomass, dry root biomass and root:shoot ratio of the two genotypes (Supplementary Data Table S2). A *t*-test was also used to evaluate daily differences in soil water moisture between genotypes from day 3 onwards. In all cases, *P* < 0.05 was considered to indicate significance. All data analyses were performed using R version 4.3.2 (R Core Team, 2022) and MATLAB (Math Works Inc., USA).

## RESULTS

### The impact of root mucilage on soil moisture depletion and transpiration dynamics

**Fig. 1.**
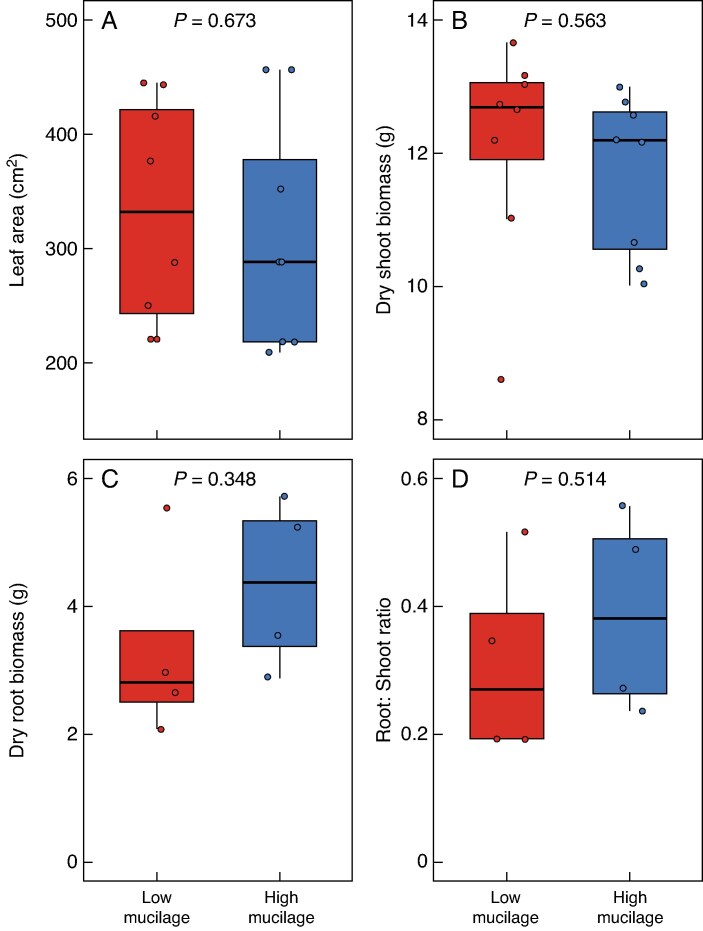
Leaf area and plant biomass for two genotypes with contrasting mucilage production. (A) Leaf area. (B) Shoot biomass. (C) Root biomass. (D) Root:shoot ratio.

**Fig. 2.**
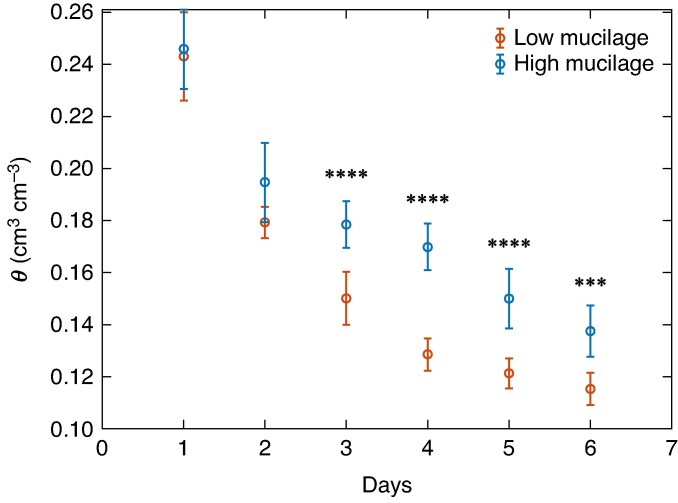
Declining soil moisture (*θ*; in centimetres cubed per centimetre cubed) over time (days after last irrigation) for low- (red) and high-mucilage (blue) genotypes. Data are mean values ± s.e. (*n* = 8). Asterisks indicate significant differences between genotypes (***P < 0.001 and ****P < 0.0001).

No significant differences were observed in leaf area between the low- (332.5 ± 34.8 cm^2^) and high-mucilage (310.95 ± 36.0 cm^2^) genotypes (*P* = 0.67, *t*-test; Fig. 1A). All values are means ± s.e. Similar results were observed in shoot biomass, root biomass and root:shoot ratio (Fig. 1B–D). Although no significant differences in leaf area and root:shoot ratio were observed, the soil moisture depletion rate during soil drying was slower in the high- compared with the low-mucilage genotype (Fig. 2). A significant difference in soil water content started from day 3 onwards (Fig. 2; *P* < 0.001).

**Fig. 3.**
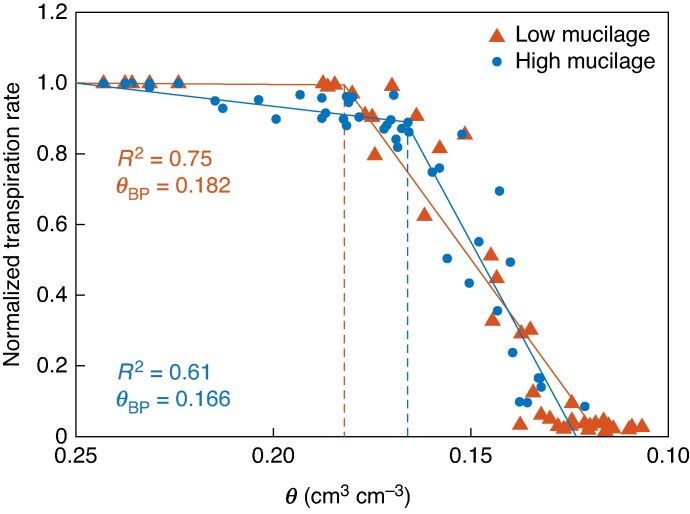
Relationship between normalized transpiration rate and volumetric soil water content (*θ*; in centimetres cubed per centimetre cubed) for low- (red) and high-mucilage (blue) genotypes at high vapour pressure deficit (2.8 kPa). The normalized transpiration rate in response to soil water content (*θ*) was analysed using a segmental linear regression model. The threshold or breakpoint (critical soil content at which plants start to downregulate their transpiration) is shown in the figure (*θ*_BP_; the dotted vertical line). The data points (filled triangles and circles) during soil drying are shown together with segmented regression lines (solid line).

During soil drying, normalized transpiration rate decreased at higher soil water content in the low-mucilage genotype [0.182 (95 % CI: 0.171–0.190) cm^3^ cm^−3^] compared with the high-mucilage genotype [0.166 (95 % CI: 0.161–0.171) cm^3^ cm^−3^], as suggested by the segmented linear regression model (Fig. 3).

### The impact of mucilage on plant response to atmospheric drying in wet soil conditions

**Fig. 4.**
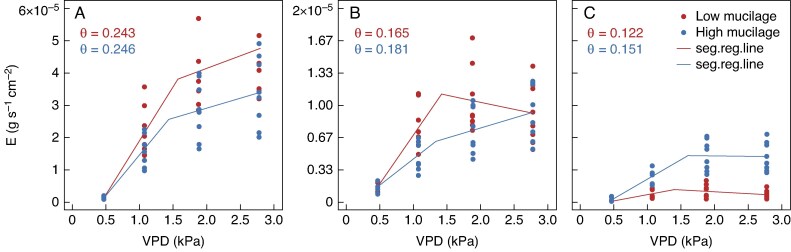
Transpiration rate (*E*; in grams per second per centimetre squared) in response to step increases in vapour pressure deficit (VPD; in kilopascals) with segmented regression lines for low- (red) and high-mucilage (blue) genotypes. (A) Wet soil: average of the first three consecutive days of transpiration rate when plants were in well-watered conditions (*n* = 8). (B) Moderate drought: average of two consecutive days of transpiration rate in the middle of the dry-down experiment (*n* = 8). (C) Severe drought: average of the last 2 days of transpiration rate during the dry-down experiment (*n* = 8). The average soil water content (*θ*) for all replicates (*n* = 8) for the respective soil condition is displayed.

In wet soil conditions, the transpiration rate increased with increasing VPD until a threshold was reached in both cowpea genotypes (Fig. 4A; Supplementary Data Fig. S2 and Table S3). Notably, in these conditions, VPD_BP_, the threshold for the restriction of transpiration rate in response to VPD, is significantly different between the two genotypes (Fig. 4A). The low-mucilage genotype exhibited a restricted transpiration rate at a significantly higher VPD (1.58 ± 0.013 kPa) than the high-mucilage genotype (1.46 ± 0.02 kPa; *P* = 0.001; Table 1; Fig. 4A). Furthermore, the slope in the relationship between transpiration rate and VPD before the breakpoint (Slope1; in grams per second per centimetre squared per kilopascal) was significantly higher in the low-mucilage genotype (0.009 ± 0.0004 g s^−1^ cm^−2^ kPa^−1^) compared with the high-mucilage genotype (0.006 ± 0.00023 g s^−1^ cm^−2^ kPa^−1^; *P* < 0.001; Table 1; Fig. 4A).

**Table 1.** Summary of the coefficient estimates and their standard errors obtained from the segmented linear regression analysis for each genotype under different soil water content.

Genotype	SWC (cm^3^ cm^−3^)	VBD_BP_ ± s.e. (kPa)	Genotypic difference	Slope1 ± s.e. (g s^−1^ cm^−2^ kPa^−1^)	Genotypic difference	Slope2 ± s.e. (g s^−1^ cm^−2^ kPa^−1^)	Genotypic difference
Low mucilage	0.243	1.58 ± 0.013	*P* = 0.001	0.009 ± 0.0004	*P* = 9.1 × 10^−5^	0.0014 ± 9.7 × 10^−5^	*P* = 0.018
High mucilage	0.246	1.46 ± 0.02		0.006 ± 0.00023		0.002 ± 0.00056	
Low mucilage	0.165	1.39 ± 0.043	*P* = 0.87	0.0033 ± 0.00012	*P* = 2.5 × 10^−8^	0.0072 ± 0.00041	*P* = 4.9 × 10^−7^
High mucilage	0.186	1.38 ± 0.051		0.0015 ± 0.00011		0.0023 ± 0.00075	
Low mucilage	0.122	1.39 ± 0.05	*P* = 0.03	0.0004 × 10^−5^ ± 8.2 × 10^−5^	*P* = 8.6 × 10^−5^	−7.09 × 10^−5^ ± 5.0 × 10^−5^	*P* = 0.048
High mucilage	0.151	1.58 ± 0.06		0.00113 × 10^−5^ ± 4.7 × 10^−5^		1.84 × 10^−5^ ± 4.1 × 10^−5^	

This includes the slopes of the first linear segment (Slope1), the vapour pressure deficit (VPD) at which the increase in transpiration with rising VPD was restricted (VPD breakpoint; VPD_BP_), and the slopes after the breakpoint (Slope2).

**Fig. 5.**
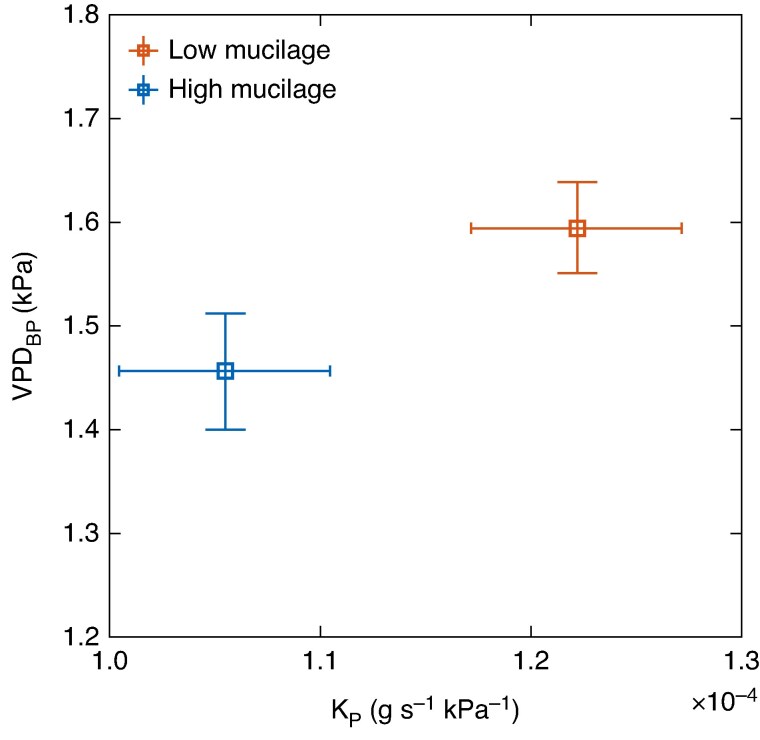
The relationship between the vapour pressure breakpoint (VPD_BP_; in kilopascals), obtained from transpiration rate–vapour pressure deficit (VPD) relationship using segmented regression analysis, and plant hydraulic conductance (*K*_P_; in grams per second per megapascal) determined in wet soil conditions for low- (red) and high-mucilage (blue) genotypes (*n* = 4).

In wet soil conditions, the low-mucilage genotype demonstrated a relatively higher VPD breakpoint (VPD_BP_ = 1.6 ± 0.048 kPa) at a higher plant hydraulic conductance (*K*_p_ = 1.22 × 10^−4^ g s^−1^ MPa^−1^) compared with the high-mucilage genotype (VPD_BP_ = 1.45 ± 0.06 kPa, at *K*_p_ = 1.0 × 10^−4^ g s^−1^ MPa^−1^; Fig. 5).

**Fig. 6.**
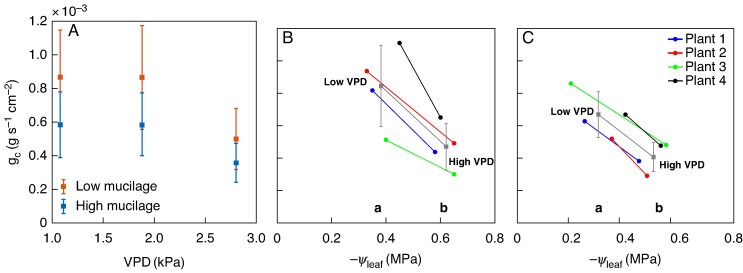
Relationship between canopy conductance ($g_{c}$; in grams per second per centimetre squared), vapour pressure deficit (VPD; in kilopascals) and leaf water potential (*ψ*_leaf_; in megapascals) in wet soil conditions. (A) Response of $g_{c\;}$ to an increase in VPD for low (red) and high-mucilage (blue) genotypes. Data are mean values ± s.e. (*n* = 4). Response of $g_{c}\;$ to a decrease in leaf water potential for low- (B) and high-mucilage (C) genotypes. Different colours represent different individual plants (*n* = 4). Grey squares with error bars (in B and C) represent the mean values and s.e. of $g_{c}$ measured at high and low VPD. Different lowercase letters indicate statistically significant differences (at *P* < 0.05) between the mean values of $g_{c}$ of the two genotypes measured at low (1.04 kPa) and high (2.8 kPa) VPD.

The response of canopy conductance ($g_{c}$) to an increase in VPD varied between low- and high-mucilage genotypes (Fig. 6A). In wet soil conditions, the low-mucilage genotype had higher canopy conductance values at all VPD levels. For instance, at low VPD (1.04 kPa), canopy conductance values were 0.87 × 10^−3^ ± 0.28 × 10^−3^ g s^−1^ cm^−2^ for the low-mucilage genotype and 0.58 × 10^−3^ ± 0.19 × 10^−3^ g s^−1^ cm^−2^ for the high-mucilage genotype. At high VPD (2.8 kPa), canopy conductance values were 0.5 × 10^−3^ ± 0.18 × 10^−3^ g s^−1^ cm^−2^ for the low-mucilage genotype and 0.35 × 10^−3^ ± 0.12 × 10^−3^ g s^−1^ cm^−2^ for the high-mucilage genotype. Regarding canopy conductance in relationship to leaf water potential, a substantial decline in canopy conductance in wet soil conditions aligned with the increase in VPD and the resulting decrease in leaf water potential in both genotypes (Fig. 6B, C). At the same VPD in wet soil conditions, the low-mucilage genotype had higher canopy conductance at more negative leaf water potential (−0.4 MPa; Fig. 6B) than the high-mucilage genotype (−0.3 MPa; Fig. 6C).

### Interaction between soil and atmospheric drying on soil–plant hydraulics

**Fig. 7.**
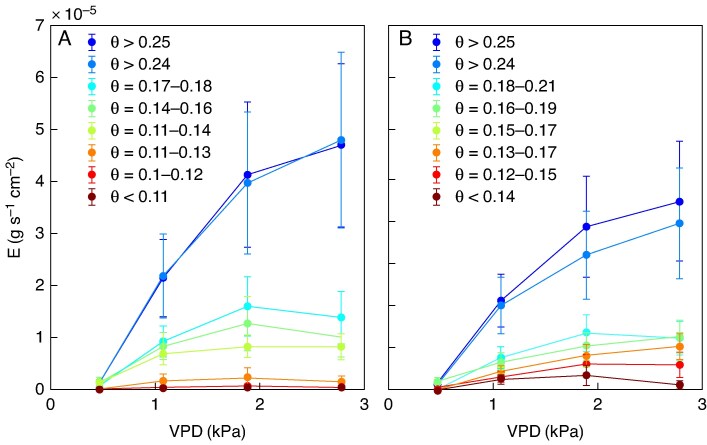
Average transpiration rate (*E*; in grams per second per centimetre squared) in response to an increase in vapour pressure deficit (VPD; in kilopascals) during soil drying. (A) Low-mucilage genotype. (B) High-mucilage genotype. Data represents mean values ± s.e. (*n* = 8). The colour represents the daily average range of soil water content (*θ*; in centimetres cubed per centimetre cubed) across all replicates.

During soil drying, the transpiration rate increased linearly with rising VPD until reaching a threshold/breakpoint in both cowpea genotypes (Figs 4B, C and 7). The low-mucilage genotype restricted transpiration rate at lower VPD (1.39 ± 0.05 kPa) than the high-mucilage genotype, which restricted transpiration rate at higher VDP (1.58 ± 0.06 kPa; *P* = 0.03; Table 1; Fig. 4C).

**Fig. 8.**
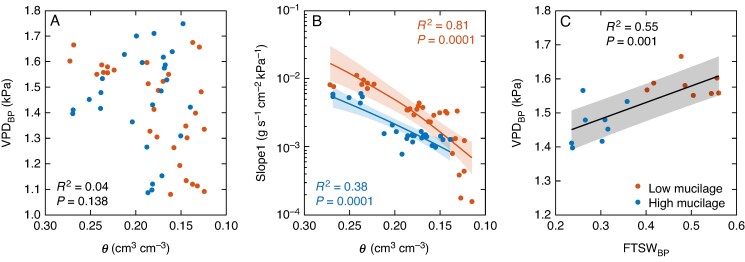
(A, B) Soil water content (*θ*; in centimetres cubed per centimetre cubed) at vapour pressure deficit breakpoints (VPD_BP_; in kilopascals) (A) and Slope1 (B). (C) Relationship between fraction of transpirable soil water breakpoints (FTSW_BP_) and VPD_BP_. The coefficient of determination (*R*^2^) and *P*-values (*P* < 0.05 considered significant) are shown. The shaded area represents the 95 % confidence interval.

In both genotypes, no clear relationship was observed between VPD_BP_, the VPD at which transpiration rate was restricted with soil drying, and soil water content (Fig. 8A). However, Slope1 became less steep with decreasing soil water content in both genotypes, with the reduction being slightly more pronounced in the low- compared with the high-mucilage genotype, although the difference was not significant (Fig. 8B). Additionally, a positive correlation was found between transpiration rate sensitivity to atmospheric drying (VPD_BP_) and transpiration rate response to soil drying (FTSW_BP_; *P* = 0.001; Fig. 8C).

**Fig. 9.**
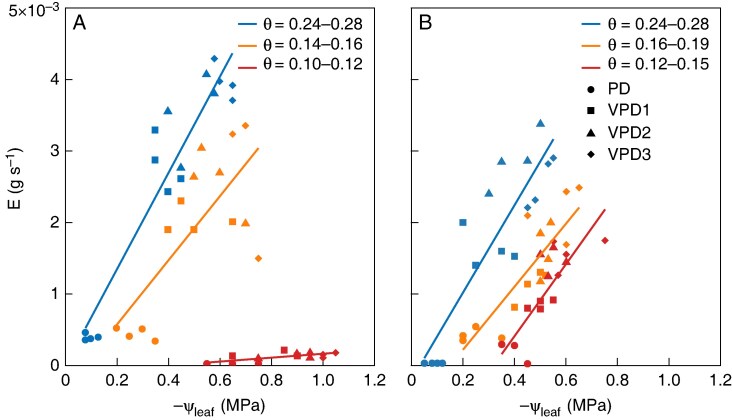
Relationship between transpiration rate (*E*; in grams per second) and leaf water potential (*ψ*_leaf_; in megapascals) at different vapour pressure deficit (VPD) levels (indicated by different shapes) at different soil water content (indicated by different colours). Each data point represents an individual replicate (*n* = 4). VPD levels were predawn (PD), low (VPD1 = 1.04 kPa), medium (VPD2 = 1.8 kPa) and high (VPD3 = 2.88 kPa) for low- (A) and high-mucilage (B) genotypes, respectively. The linearly fitted slope of the *E*–*ψ*_leaf_ relationship equals the soil–plant hydraulic conductance (in grams per second per megapascal).

In wet soil, the transpiration rate at high VPD was slightly higher in the low-mucilage genotype than in the high-mucilage genotype (Fig. 9A). As the soil dried, the transpiration rate in the low-mucilage genotype dropped faster (Fig. 9A) compared with the high-mucilage genotype (Fig. 9B). Leaf water potential decreased with an increasing VPD in wet and severe drought conditions (Fig. 9). The high-mucilage genotype generally exhibited less negative mean leaf water potentials than the low-mucilage genotype (Fig. 9). In wet soil conditions and high VPD, the higher negative leaf water potential was −0.51 ± 0.046 MPa for the high-mucilage genotype and −0.62 ± 0.036 MPa for the low-mucilage genotype. In severe drought conditions, the most negative leaf water potential was −1.0 ± 0.04 MPa for the low-mucilage genotype under high VPD, while the high-mucilage genotype had a less negative water potential of −0.61 ± 0.009 MPa in the same conditions (Fig. 9).

## DISCUSSION

We investigated the regulation of plant water use of two cowpea genotypes with contrasting mucilage production during combined edaphic and atmospheric stress. In wet soil conditions, the low-mucilage genotype had a higher VPD breakpoint (a measure of transpiration restriction) than the high-mucilage genotype. The initial slope of transpiration rate in response to VPD (Slope1; here interpreted as the maximum conductance; Sinclair *et al.*, 2008; Sadok and Sinclair, 2010; Schoppach *et al.*, 2017) was significantly higher in the low-mucilage genotype compared with the high mucilage genotype in wet soil conditions. On the contrary, during soil drying, the transpiration rate declined earlier in the low-mucilage genotype compared with the high-mucilage genotype, supporting the hypothesis that mucilage helps to maintain the hydraulic continuity between roots and soil at lower water potentials in the high-mucilage genotype. Furthermore, the high-mucilage genotype exhibited less negative mean leaf water potentials than the low-mucilage genotype.

### Impacts of mucilage on regulation of water fluxes in wet soils

Despite similar atmospheric demand scenarios, comparable leaf area and root:shoot ratio, significant differences in soil water depletion rate between the two genotypes highlight the role of mucilage in regulating water fluxes at the root–soil interface, even in ample water conditions. In previous studies, mucilage has been shown to influence the viscosity of soil solution, thereby reducing the saturated hydraulic conductivity (Ahmed *et al.*, 2018*a*, *b*; Kroener *et al.*, 2018; Benard *et al.*, 2019; Landl *et al.*, 2021). The reduced saturated hydraulic conductivity might affect water flow at the root–soil interface, thereby decreasing transpiration at the plant scale (Berauer *et al.*, 2023; Abdalla *et al.*, 2024). The underlying mechanism is that in well-watered conditions, mucilage forms a viscous polymeric layer at the root–soil interface (Kroener *et al.*, 2014; Volk *et al.*, 2016), which increases the viscosity of the liquid phase in the rhizosphere, reducing saturated hydraulic conductivity (Ahmed *et al.*, 2018*b*). This could limit water supply to the roots under increasing evaporative demand. This could be a plausible explanation for the slightly slower rate of soil moisture depletion observed in the high-mucilage genotype compared with the low-mucilage genotype (Fig. 2).

Interestingly, the high-mucilage genotype exhibited a lower VPD breakpoint in transpiration rate response to rising VPD (Fig. 4). This earlier breakpoint (Table 1; Fig. 4A; Supplementary Data Fig. S2) and the associated lower maximum soil–plant conductance (Table 1; Fig. 4A) might be linked to a reduced water supply to the roots under increasing evaporative demand, potentially owing to the mucilage-induced decline in saturated hydraulic conductivity, as described above. Furthermore, we observed that lower plant conductance was correlated with a lower VPD breakpoint, whereas higher plant conductance was associated with a higher VPD breakpoint (Fig. 5). A previous study also showed that a low VPD breakpoint is associated with low plant hydraulic conductance (Choudhary *et al.*, 2014), hence plants with higher hydraulic conductance might exhibit a delayed response to increasing VPD (e.g. reaching a higher transpiration rate before breakpoints occur). In contrast, Koehler *et al.* (2024) reported that C_4_ cereals (sorghum, maize and millet) showed earlier restrictions in transpiration rate at lower VPD when their maximum conductance (Slope1) was high in low-VPD conditions. The authors suggested that higher maximum conductance at low VPD might predispose plants to earlier regulation of transpiration as evaporative demand increases. Therefore, in wet soil conditions, we assume that transpiration rate restrictions in response to VPD might not depend solely on plant conductance, but might also be impacted by mucilage-induced changes in rhizosphere hydraulic properties, because mucilage can affect water flow to the roots, especially under high transpiration demand (Ahmed *et al.*, 2018*a*, *b*; Benard *et al.*, 2019; Abdalla *et al.*, 2024).

### Impacts of mucilage on water flux regulation during severe drought

It has been shown that mucilage decreases the saturated hydraulic conductivity of soils by several orders of magnitude (Ahmed *et al.*, 2018*a*, *b*; Benard *et al.*, 2019; Zarebanadkouki *et al.*, 2019), but also enhances the water content in the rhizosphere compared with the bulk soil at low soil moisture (Carminati *et al.*, 2010). This effect would attenuate the gradient in matric potential at the root–soil interface, thereby facilitating root water uptake during soil drying (Carminati *et al.*, 2010; Ahmed *et al.*, 2014; Kroener *et al.*, 2014; Abdalla *et al.*, 2024). Simulation studies showed that the high water-holding capacity of mucilage in the rhizosphere delays the onset of stress and supports sustained transpiration (Schwartz *et al.*, 2016; Landl *et al.*, 2021). Our study further supports the hypothesis that, during soil drying, the genotype with higher mucilage production exhibits less negative leaf water potential than the low-mucilage genotype (Fig. 9B). This might be attributable exclusively to the presence of mucilage, which attenuates the gradients in water potential in the rhizosphere (Carminati *et al.*, 2017; Ahmed *et al.*, 2018*a*). Recently, Abdalla *et al.* (2024) investigated the same cowpea genotypes and revealed that the genotype with high mucilage production showed a less steep decline in water potential near the root surface in comparison to the genotype with low mucilage production. This suggests that mucilage plays a crucial role in delaying the onset of hydraulic limitations in drying soil (Ahmed *et al.*, 2018*b*).

### Impacts of mucilage on water flux regulation under increasing VPD

In wet soil conditions, the high-mucilage genotype showed a gradual increase in transpiration rate (Fig. 4A; Supplementary Data Fig. S2) and an earlier restriction in transpiration rate with increasing VPD (i.e. lower VPD_BP_; Table 1; Fig. 4A) compared with the low-mucilage genotype. The lower initial slope and earlier stomatal response led to less overall water loss through transpiration in higher VPD conditions. This highlights that mucilage decreases the saturated hydraulic conductivity in wet soil conditions, which potentially slows down root water uptake, thereby conserving soil moisture for critical phases, such as flowering and grain filling (Ahmed *et al.*, 2018*a*). Conversely, Jafarikouhini *et al.* (2022) reported that in sweet corn, the VPD_BP_, the VPD at which transpiration becomes restricted, was negatively correlated with Slope1 (maximum canopy conductance). Likewise, Koehler *et al.* (2024) found the same relationship in maize, pearl millet and sorghum. These variations might be attributable to differences in water potential gradient in the rhizosphere, which might explain the earlier onset of restrictions of transpiration rate at lower VPD.

Plants can more easily sustain reduced fluxes during soil drying for extended periods when the daily average transpiration rate is lower, owing to the restricted transpiration rate during lower VPD conditions (Koehler *et al.*, 2023). Vadez (2014) highlighted that limiting the transpiration rate at lower VPD can increase daily transpiration efficiency and that restricting the transpiration rate in the early stages of the soil drying cycle can lead to a conservative seasonal transpiration response to soil drying. This strategy enhances water use during critical reproductive stages of crop development and yield stability in water stress conditions (Gholipoor *et al.*, 2013; Vadez, 2014). Schoppach *et al.* (2014) found that a drought-tolerant wheat breeding line displayed a conservative behaviour by limiting its transpiration rate with increasing VPD, effectively saving soil water moisture for later use. Our results show that the low-mucilage genotype also exhibits a more conservative approach on a ‘seasonal basis’ by limiting transpiration at higher FTSW_BP_, whereas the high-mucilage genotype displays an anticipatory behaviour by being more conservative on a daily basis by limiting transpiration at relatively low VPD_BP_ (Fig. 8C). Similar findings in soybean (Devi *et al.*, 2014) and maize (Gholipoor *et al.*, 2013) suggest that a limited transpiration rate in response to increased VPD initiates a decline in transpiration at lower FTSWs. This conservation of soil water by restricting transpiration rates can improve agricultural sustainability and resilience to climate change, maximizing crop yields and optimizing plant water use.

Although we have shown that root mucilage might impact plant water use during both edaphic and atmospheric stress, it is essential to recognize other root traits, e.g. differences in anatomical, architectural and/or axial and radial conductivity, that also determine the capacity of root systems in root water uptake (e.g. Baca Cabrera *et al.*, 2024). We observed no significant differences in root biomass or in root:shoot ratio between the two genotypes (Fig. 1C, D). Furthermore, Abdalla *et al.* (2024) found no significant differences in root length and diameter between the same two cowpea genotypes grown in loamy soil. The authors showed a significant decline in water potential across the rhizosphere in the low-mucilage genotype, whereas the high-mucilage genotype attenuated the decline in water potential across the rhizosphere (Abdalla *et al.*, 2024). These findings are in line with our results, which indicate that mucilage plays a crucial role in enhancing root water uptake, especially in drying soils. Moreover, although root mucilage might modulate plant water use in both atmospheric and edaphic drought conditions, this advantage might be associated with a trade-off. For instance, increased mucilage production entails a cost in assimilated carbon, potentially diverting resources away from root development and reproductive processes (Lynch, 2007; Raza *et al.*, 2024). Furthermore, the influence of environmental conditions on mucilage exudation rates between the two genotypes remains unknown. Further research is needed to elucidate how factors such as climate and soil type affect mucilage exudation.

### Conclusion

The collective interplay between edaphic and atmospheric drought is a topic of growing interest in the scientific community (Liu *et al.*, 2020; Sadok *et al.*, 2021; Gleason *et al.*, 2025). In this study, we investigated two cowpea genotypes differing in mucilage production under increasing VPD and progressive soil drying. We showed that as VPD increases, plant water use is regulated through a reduction in maximum plant conductance and genotype-specific thresholds for transpiration restriction. Root mucilage is thus suggested as a beneficial trait for enhancing plant water use both on a daily basis and over extended periods during soil drying. Further investigation into the genetic and physiological mechanisms underlying plant response to VPD could guide breeding efforts to develop crops with enhanced traits, such as root mucilage production.

## Supplementary Material

mcaf182_Supplementary_Data

## SUPPLEMENTARY DATA

Supplementary data are available at *Annals of Botany* online and consist of the following. Figure S1: soil water retention (A) and hydraulic conductivity (B) curves of sandy soil used for this experiment. The fitting parameters of the water retention and hydraulic conductivity curves were estimated using Brooks and Corey model. Figure S2: average transpiration rate (*E*; in grams per second per centimetre squared) for all replicates (*n* = 8) in response to step increases in vapour pressure deficit (VPD; in kilopascals) with segmented regression analysis for low- (red) and high-mucilage (blue) genotypes in well-watered conditions over two consecutive days before the start of the dry-down experiment. Table S1: climate chamber program used for cowpea plant experiments in sandy soil. Table S2: outcome of statistical analysis of VPDBP, slope1, leaf area, dry shoot and root biomass and root:shoot ratio. Table S3: slopes, VPD breakpoints, and other parameters obtained from the *E*–VPD relationship using segmented regression model for replicates before the dry-down experiment (in wet conditions). The grey-shaded rows represent the average of 2 days.
