# Supplementary material for: Root mucilage enhances plant water use under combined soil and atmospheric drought
Source: Ann Bot. 2025 Aug 13;136(5-6):1131–42. doi: 10.1093/aob/mcaf182 (PMC12682819; doi:10.1093/aob/mcaf182)
Supplement: mcaf182_Supplementary_Data [file mcaf182_supplementary_data.zip › Revised_Supplementary tables_AOB_2025_134.pdf]

Supplementary tables

Table S1: Climate chamber program used for cowpea plant experiments in sandy soil.

Segment	VPD Levels	Start time	End time	Duration	RH [%]	T [°C]	VPD [kPa]
1	VPD1 Ramp	09:00	09:30	00:30			
2	VPD1	09:30	12:30	03:00	59	23.5	1.1
3	VPD2 Ramp	12:30	13:00	00:30			
4	VPD2	13:00	16:00	03:00	52	29.5	1.8
5	VPD3 Ramp	16:00	16:30	00:30			
6	VPD3	16:30	19:30	03:00	52	34.5	2.78
7	Cooling Ramp	19:30	20:00	00:30			
8	Cooling	20:00	23:00	03:00	60	22	1.1
9	Night Ramp	23:00	23:30	00:30			
10	Night	23:30	09:00	09:30	20	80	0.5

Table S2: Outcome of statistical analysis of VPDBP, slope1, leaf area, dry shoot and root biomass and root:shoot ratio.

Tested parameter	Measurements normally distributed?	Homogeneity of variances?	Applied test	p-value
VPD_{BP}	yes	yes	Independent samples t- test	0.001
Slope1	yes	yes	Independent samples t- test	0.0001
Dry shoot biomass	yes	yes	Independent samples t- test	0.56
Leaf area	yes	yes	Independent samples t- test	0.67
Root:shoot ratio	yes	yes	Independent samples t- test	0.5136
Dry root biomass	yes	yes	Independent samples t- test	0.07369
Soil moisture on each day from day 3 on	yes	yes	Independent samples t- test	< 0.001

Table S3: Slopes, VPD breakpoints, and other parameters obtained from the E~VPD relation using segmented regression model for replicates before the dry-down experiment (under wet conditions). The grey-shaded rows represent the average of two days.

First day (Supplementary Fig. S4A)				
Genotype	Slope1 (g s^{-1} cm^{-2} kPa^{-1})	Slope2 (g s^{-1} cm^{-2} kPa^{-1})	VPD_{BP} (kPa)	VPD_{BP} (SE) (kPa)
Low Mucilage	0.010592	0.0198	1.630759	0.135568
High Mucilage	0.007192	0.0128	1.541802	0.110881
Second day (Supplementary Fig. S4B)				
Low Mucilage	0.01088	0.0188	1.506058	0.146553
High Mucilage	0.006758	0.0116	1.319758	0.137966
Average (low mucilage)	0.01088	0.0193	1.5688	0.14106
Average (High mucilage)	0.006975	0.0122	1.4316	0.1244
