# Supplementary material for: Root mucilage enhances plant water use under combined soil and atmospheric drought
Source: Ann Bot. 2025 Aug 13;136(5-6):1131–42. doi: 10.1093/aob/mcaf182 (PMC12682819; doi:10.1093/aob/mcaf182)
Supplement: mcaf182_Supplementary_Data [file mcaf182_supplementary_data.zip › Revised_supplementary_AOB_2025_134_Figure_S1.pdf]

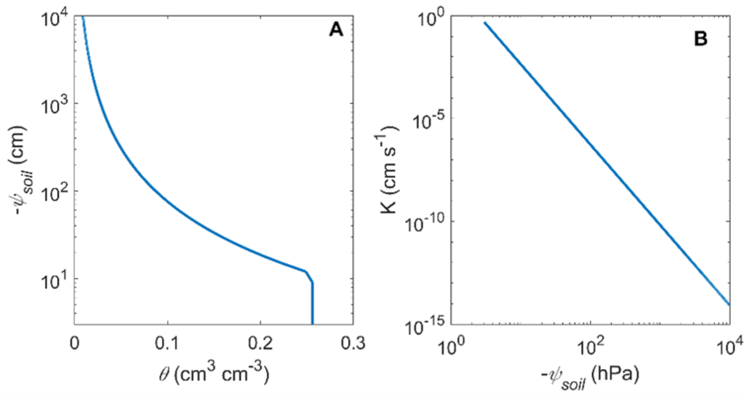

Fig. S1: Soil water retention (A) and hydraulic conductivity (B) curves of sandy soil used for this experiment. The fitting parameters of the water retention and hydraulic conductivity curves were estimated using Brooks and Corey model.
