# Supplementary material for: Root mucilage enhances plant water use under combined soil and atmospheric drought
Source: Ann Bot. 2025 Aug 13;136(5-6):1131–42. doi: 10.1093/aob/mcaf182 (PMC12682819; doi:10.1093/aob/mcaf182)
Supplement: mcaf182_Supplementary_Data [file mcaf182_supplementary_data.zip › Revised_supplementary_AOB_2025_134_Figure_S2.pdf]

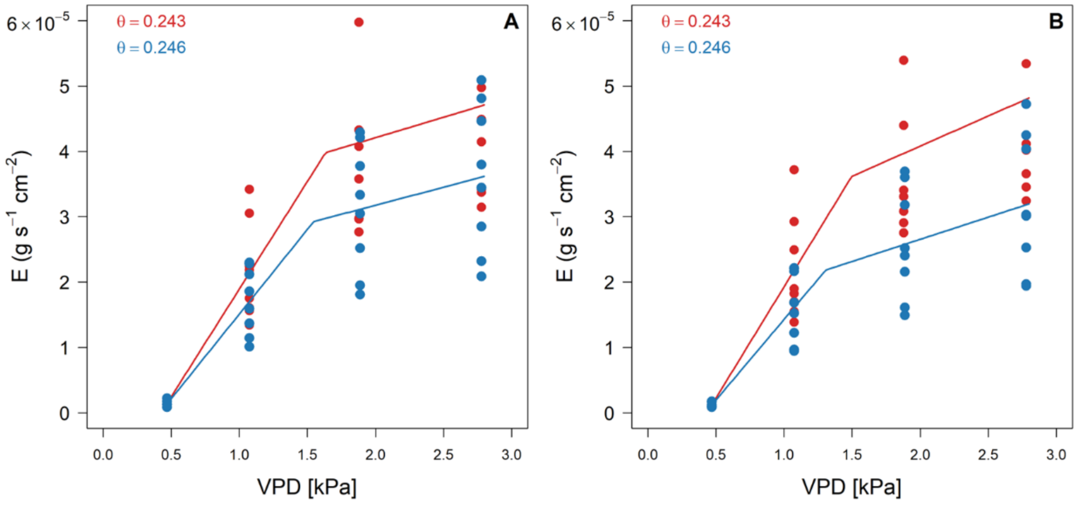

Fig. S2: Average transpiration rate (E; g s-1 cm-2) for all replicates (n = 8) in response to step increases in vapor pressure deficit (VPD, kPa) with segmented regression analysis for low (red) and high (blue) mucilage genotypes under well-watered conditions over two consecutive days before the start of the dry-down experiment.
